# Knowledge distillation for named entity recognition in traditional chinese medicine

**DOI:** 10.1038/s41598-026-56313-y

**Published:** 2026-06-08

**Authors:** Wangping Xiong, Hongda Huang, Yingjun Yang, Ming Yang, Xiang Zhou, Shaobo Chen, Xin Cheng, Xian Zhou, Zhaoxing Xu

**Affiliations:** 1 School of Intelligent Medicine and Information Engineering, Jiangxi University of Chinese Medicine, Nanchang, 330004 China; 2 Computer College, Jiangxi Institute of Fashion Technology, Nanchang, 330201 China

**Keywords:** Computer science, Data processing

## Abstract

Named entity recognition (NER) in traditional Chinese medicine (TCM) text is central to the structuring and intelligent application of TCM knowledge. TCM case texts are characterized by sparse entity distributions and limited domain-specific data. Existing methods often struggle to recognize low-frequency entities and show limited capacity for effective knowledge transfer. This paper proposes a TCM NER framework incorporating structured knowledge distillation. First, multi-source TCM corpora are constructed using natural language processing techniques. A teacher model is trained on high-density structured data, and its semantic knowledge is transferred to the student model through soft labels. The student model uses BERT for encoding, BiLSTM for sequential feature extraction, Transformer for global context modeling, and CRF for structured sequence decoding. Experimental results on the constructed StudentDataset show that the distilled TBTC model improved Precision by 8.01 percentage points, Recall by 5.31 percentage points, and F1-score by 6.67 percentage points over its non-distilled counterpart, achieving an overall F1-score of 77.01.

## Introduction

Structuring TCM knowledge is essential for transforming empirical wisdom into computable and analyzable data^1^. With the rapid advancement of artificial intelligence (AI) technologies, the digitalization and structural organization of voluminous and intricate TCM knowledge systems has emerged as a focal point in contemporary research^2^. As a core NLP task, NER supports the intelligent use of TCM knowledge by identifying domain-specific terms and facilitating knowledge graph construction. It provides structured support for extracting key medical entities, including herbs, syndromes, symptoms, and formulas, thereby promoting the AI-driven use of TCM knowledge^3^.

The NER task in TCM confronts multiple domain-specific challenges. TCM texts often contain sparse entity distributions and limited domain-specific data. Many entities appear only in specialized historical literature, which leads to insufficient training samples^4^. Furthermore, TCM terminology exhibits significant polysemy, requiring contextual interpretation for accurate comprehension. Domain-specific terms such as “Xingwei (property and flavor)” and “Guijing (meridian tropism)” demonstrate meaning variability across different therapeutic formulations or symptom contexts, thereby amplifying text processing complexity^5^. In recent years, deep learning architectures including BERT and BiLSTM have demonstrated robust performance across diverse domains. BERT leverages pre-trained language models to generate context-aware representations, while BiLSTM effectively captures long-range dependencies within sequential data, both showing promising efficacy in TCM NER tasks^6–8^. However, existing methodologies inadequately address the unique linguistic characteristics of TCM texts, failing to account for the semantic complexity of TCM terminology, which leads to persistent difficulties in low-frequency entity recognition^9,10^.

To address these challenges, this study proposes TCMBERT-BiLSTM-Transformer-CRF (TBTC), a NER framework integrating structure-aware knowledge distillation for TCM. Initially, we constructed a multi-source TCM corpus encompassing herbological data, therapeutic formulations, and clinical case records, thereby establishing a comprehensive data infrastructure for TCM research. Subsequently, a teacher model was trained on high entity-density structured data, leveraging its enriched semantic knowledge to transfer domain expertise to the student model through soft label distillation. In particular, the proposed framework incorporates structured knowledge from two complementary aspects: (1) implicit structural patterns learned by the teacher model from high-density entity corpora, and (2) explicit sequence-level constraints modeled by the CRF layer in the student model. The student model combines BERT, BiLSTM, and Transformer to capture contextual, sequential, and global features of complex TCM terminology. Finally, a CRF layer performs structured sequence decoding, improving entity boundary detection precision and categorical consistency. The principal contributions of this work are threefold:

- We have constructed a multi-source TCM corpus, providing annotated linguistic resources for TCM NER tasks, thereby addressing the persistent challenge of data scarcity in TCM textual analysis.
- We propose a TCMBERT-BiLSTM-Transformer-CRF model to better address term polysemy in TCM texts.
- We design a knowledge distillation framework to improve recognition performance for low-frequency entities through teacher–student knowledge transfer.

## Related work

Recent years have witnessed extensive research on Chinese NER, with deep pre-trained language models emerging as the predominant approach. The architectural framework integrating BERT with Bidirectional Long Short-Term Memory (BiLSTM) and Conditional Random Field (CRF) has been empirically validated as an effective solution^11^. Li et al. pioneered the application of BERT to Chinese clinical NER tasks, establishing its feasibility in medical contexts through domain-specific pre-training on large-scale clinical corpora combined with BiLSTM-CRF architecture and medical lexicon feature augmentation^12^. Concurrently, to address structurally complex medical texts characterized by severe semantic nesting, recent studies have introduced advanced global modeling architectures and nested entity recognition mechanisms. Notably, researchers developed a TCM-oriented NER framework incorporating RoBERTa-wwm-ext-large for lexical semantic representation, BiLSTM for contextual dependency encoding, and GlobalPointer for precise nested entity modeling. The RoBGP (RoBERTa-BiLSTM-GlobalPointer) architecture performs well in TCM NER, particularly for complex terminology and hierarchical entities^13^.

Beyond continuous optimization of structural modeling, knowledge distillation strategies for NER tasks have garnered substantial research attention in recent years^14^. Initial research efforts concentrated on probability distribution distillation via Softmax output layers, where soft labels generated by teacher models provided semantic guidance for student models to mitigate performance degradation of lightweight models under low-resource conditions. Subsequently, studies expanded the distillation scope by incorporating intermediate feature-level and attention weight-level knowledge transfer mechanisms, achieving fine-grained knowledge propagation across neural architectures. These advancements have demonstrated marked efficacy in NER applications spanning legal, financial, and biomedical domains^15^. Concurrently, innovative approaches integrating knowledge distillation with structured decoders have been proposed, effectively enhancing student models’ boundary detection precision and structural awareness in sequence labeling tasks^16^.

Despite notable progress in TCM NER through deep pre-trained models, current methodologies exhibit persisting limitations. Existing frameworks inadequately address the inherent polysemy and complex contextual dependencies of TCM terminologies, constraining model performance in capturing the dynamic semantic variations characteristic of domain-specific expressions. Furthermore, while knowledge distillation techniques have demonstrated substantial efficacy in cross-domain NER tasks, their application to TCM textual analysis remains underdeveloped. Prevailing approaches fail to incorporate the unique ontological structure of TCM knowledge systems, lacking robust domain-specific knowledge transfer mechanisms to effectively handle challenges including sparse entity distribution, domain-specific data scarcity, and terminological polysemy that permeate TCM corpora.

## Methods

**Fig. 1:**
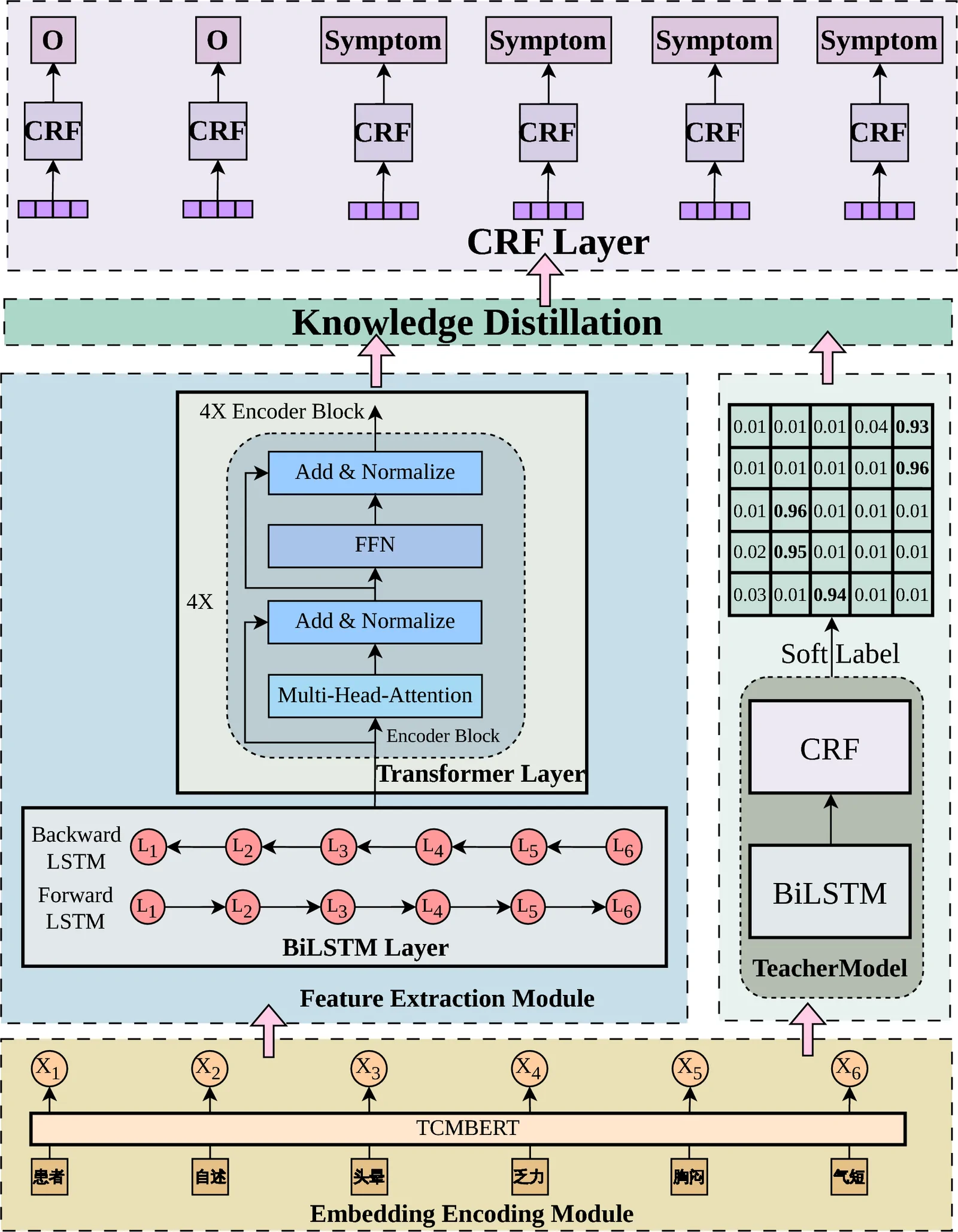
Architecture of TBTC.

The proposed TBTC framework consists of several modules, as shown in Figure 1. The embedding encoder module initially transforms TCM textual inputs into dense contextualized feature vectors. Subsequently, the feature extraction module synergistically integrates BiLSTM layers and Transformer blocks to capture both sequential patterns and task-specific global contextual dependencies. In particular, the BiLSTM layer enhances order-sensitive contextual modeling, while the additional Transformer layers operate on the BiLSTM-enhanced representations to model long-distance token interactions relevant to downstream sequence labeling. The knowledge distillation module transfers informative token-level class distributions from the teacher model to the student model through soft label alignment. Finally, a CRF layer performs structured sequence decoding to optimize entity boundary detection and categorical classification. Prior to model training, the input data are processed through a preprocessing pipeline involving rigorous data cleaning, domain-specific tokenization, and expert-validated annotation, thereby improving the consistency and quality of the training data.

In the proposed framework, the teacher model is first trained on an independent high entity-density annotated dataset that follows the same annotation criteria and label schema as the student training data. Compared with the full student dataset, this teacher dataset contains more entity instances per sentence and richer class co-occurrence patterns. As a result, the dominance of the O class is alleviated, and the resulting supervision provides more informative token-level class distribution signals. The student model is then trained on the full dataset under the joint supervision of gold labels and teacher-provided soft targets. Although the teacher model is architecturally simpler than the student model, it serves as an informative auxiliary supervisory source because it is trained on data with denser entity annotations. In our setting, a more complex teacher did not yield better distillation performance.

### Embedding encoder module

**Fig. 2:**
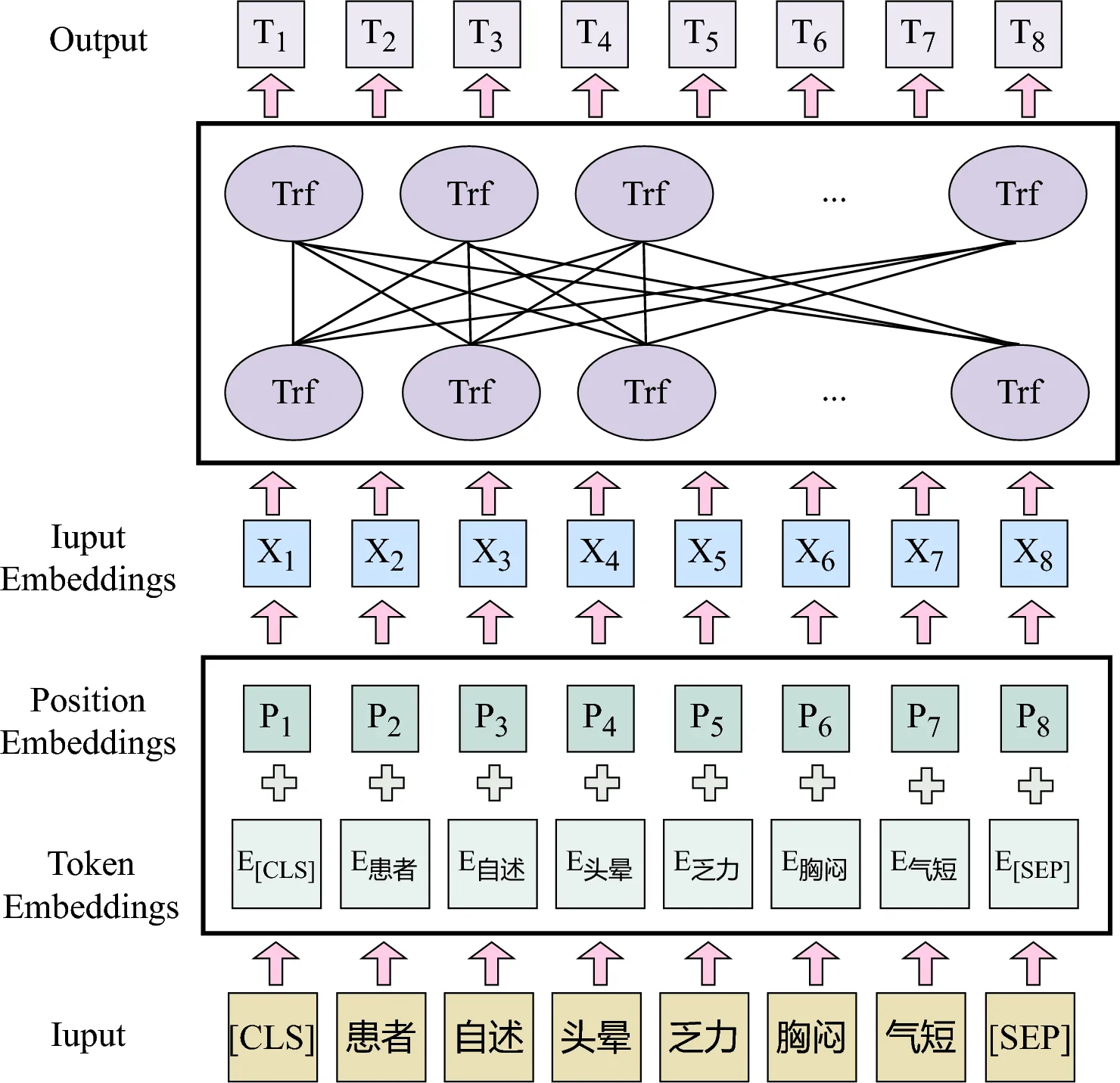
Architecture of TCMBERT.

As depicted in Figure 2, the TBTC model employs the TCMBERT (Traditional Chinese Medicine BERT) architecture for embedding encoding. TCMBERT is a domain-specific pre-trained language model trained on large-scale unannotated TCM corpora to capture domain-specific semantic representations^17^. The input text undergoes tokenization and is mapped to corresponding token vectors via the embedding layer. Each token $w_t$ is transformed into a vector $\textbf{e}_t \in \mathbb {R}^d$ through the token embedding matrix $\textbf{E}$, combined with positional encoding $\textbf{p}_t$, yielding the final input representation:

1 $$\begin{aligned} \textbf{h}_t^{(0)} = \textbf{e}_t + \textbf{p}_t \end{aligned}$$

Special tokens [CLS] and [SEP] are appended to each sentence, with [PAD] tokens added for sequence length normalization. During BERT-style pre-training, the model utilizes a Masked Language Model (MLM) objective, where a randomly selected subset of input tokens $\mathcal {M} = \{w_{m_1}, \ldots , w_{m_k}\}$ is masked. The corresponding loss function is defined as:

2 $$\begin{aligned} \mathcal {L}_{\text {MLM}} = -\sum _{w_i \in \mathcal {M}} \log P(w_i \mid \textbf{h}_{\setminus i}) \end{aligned}$$

where $\textbf{h}_{\setminus i}$ denotes contextual representations excluding the *i*-th token. To prevent model overfitting to specific masking patterns, a stochastic masking strategy is implemented: 80% probability of replacing selected tokens with [MASK], 10% with random tokens, and 10% retaining original tokens. Through iterative optimization minimizing $\mathcal {L}_{\text {MLM}}$, the TCMBERT encoder generates contextualized token representations:

3 $$\begin{aligned} \textbf{H} = [\textbf{h}_1, \textbf{h}_2, \ldots , \textbf{h}_n] \end{aligned}$$

where $\textbf{H} \in \mathbb {R}^{n \times d}$ represents the sequence of contextual embeddings for all input tokens, serving as input to the subsequent feature extraction module.

### Feature extraction module

#### BiLSTM layer

The bidirectional long short-term memory (BiLSTM) network is employed to capture order-sensitive contextual information in textual sequences and to strengthen sequential dependency modeling^18^. As an extension of conventional LSTM networks^19^, BiLSTM enhances contextual representation by modeling the input sequence in both forward and backward directions.

Given an input sequence $\textbf{X} = [\textbf{x}_1, \textbf{x}_2,..., \textbf{x}_n]$ where $\textbf{x}_i \in \mathbb {R}^{d}$ denotes the *i*-th token embedding, the contextual embeddings generated by TCMBERT are directly used as BiLSTM inputs, i.e., $\textbf{x}_i=\textbf{h}_i$. The BiLSTM processes sequential information through dual directional computations:

1. **Forward LSTM**: Processes left-to-right sequence flow:
  4 $$\begin{aligned} \overrightarrow{\textbf{h}}_i = \textrm{LSTM}_{\textrm{fwd}}(\textbf{x}_i, \overrightarrow{\textbf{h}}_{i-1}) \end{aligned}$$
2. **Backward LSTM**: Processes right-to-left sequence flow:
  5 $$\begin{aligned} \overleftarrow{\textbf{h}}_i = \textrm{LSTM}_{\textrm{bwd}}(\textbf{x}_i, \overleftarrow{\textbf{h}}_{i+1}) \end{aligned}$$
3. **Bidirectional Output**: Concatenates hidden states:
  6 $$\begin{aligned} \textbf{h}_i^{\mathrm {(bi)}} = [\overrightarrow{\textbf{h}}_i ; \overleftarrow{\textbf{h}}_i] \in \mathbb {R}^{2d_h} \end{aligned}$$

The resultant sequence is denoted as

7 $$\begin{aligned} \textbf{H}^{\mathrm {(bi)}} = [\textbf{h}_1^{\mathrm {(bi)}}, ..., \textbf{h}_n^{\mathrm {(bi)}}], \end{aligned}$$

which preserves the original sequence structure while incorporating bidirectional contextual features. The BiLSTM hidden size is set such that the bidirectional output dimension matches the Transformer input dimension, i.e., $2d_h = d$.

To improve training stability and maintain feature distribution consistency, layer normalization is applied:

8 $$\begin{aligned} \widetilde{\textbf{H}}^{(0)} = \textrm{LayerNorm}(\textbf{H}^{\mathrm {(bi)}}) \end{aligned}$$

The normalized sequence $\widetilde{\textbf{H}}^{(0)}$ serves as the input to subsequent Transformer layers.

**Fig. 3:**
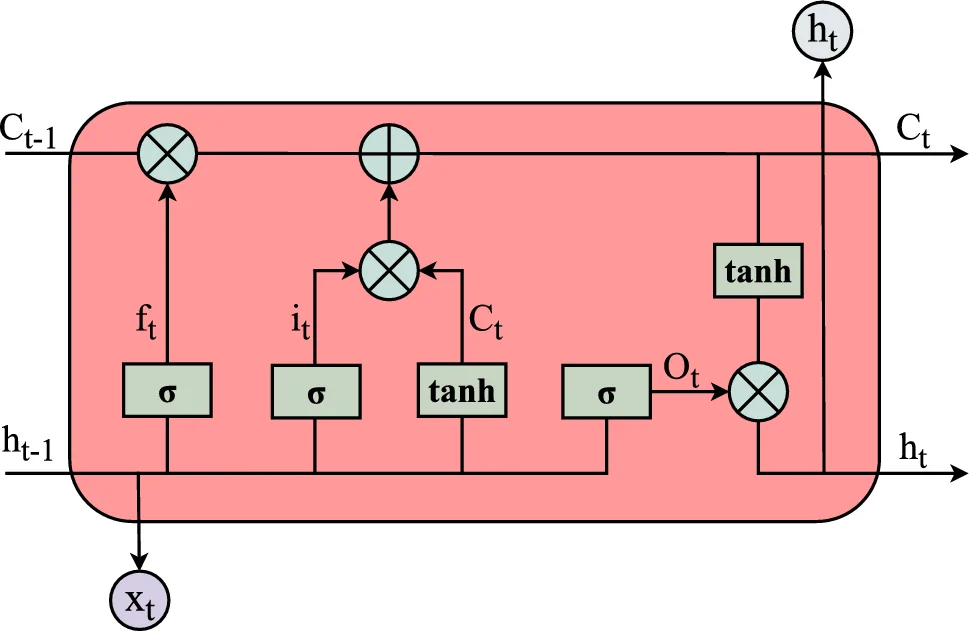
Architecture of LSTM cell.

As illustrated in Figure 3, each LSTM cell^20^ implements gating mechanisms as follows:

9 $$\begin{aligned} \text {Forget Gate:} \quad&\textbf{f}_t = \sigma (\textbf{W}_f [\textbf{h}_{t-1}, \textbf{x}_t] + \textbf{b}_f) \end{aligned}$$

10 $$\begin{aligned} \text {Input Gate:} \quad&\textbf{i}_t = \sigma (\textbf{W}_i [\textbf{h}_{t-1}, \textbf{x}_t] + \textbf{b}_i) \end{aligned}$$

11 $$\begin{aligned} \text {Candidate Memory:} \quad&\tilde{\textbf{C}}_t = \tanh (\textbf{W}_C [\textbf{h}_{t-1}, \textbf{x}_t] + \textbf{b}_C) \end{aligned}$$

12 $$\begin{aligned} \text {Memory Update:} \quad&\textbf{C}_t = \textbf{f}_t \odot \textbf{C}_{t-1} + \textbf{i}_t \odot \tilde{\textbf{C}}_t \end{aligned}$$

13 $$\begin{aligned} \text {Output Gate:} \quad&\textbf{o}_t = \sigma (\textbf{W}_o [\textbf{h}_{t-1}, \textbf{x}_t] + \textbf{b}_o) \end{aligned}$$

14 $$\begin{aligned} \text {Hidden State:} \quad&\textbf{h}_t = \textbf{o}_t \odot \tanh (\textbf{C}_t) \end{aligned}$$

where $\sigma (\cdot )$ denotes the sigmoid activation function, *⊙* denotes element-wise multiplication, $\textbf{C}_t$ represents the cell state, and $\textbf{h}_t$ is the hidden state at time step *t*.

By integrating information from both forward and backward directions, the BiLSTM layer effectively captures order-sensitive contextual dependencies in TCM text and provides enriched sequential representations for the subsequent Transformer encoder blocks.

#### Transformer layer

**Fig. 4:**
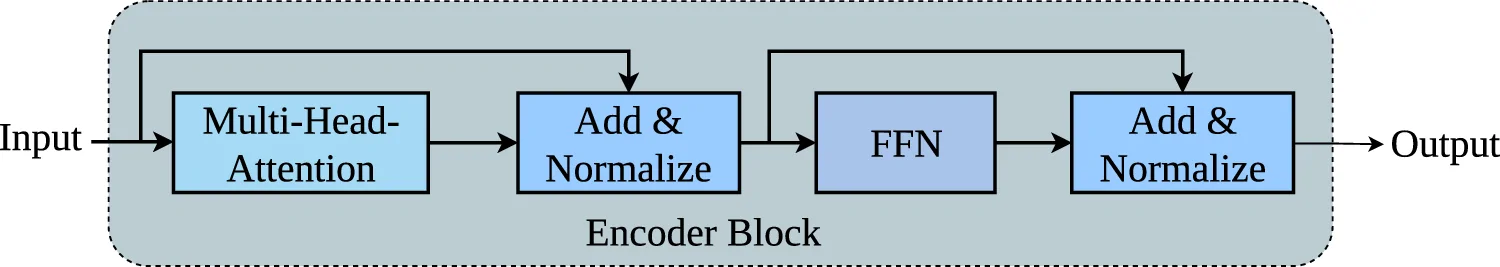
Architecture of the transformer encoder block.

To further capture long-range dependencies and enhance task-specific global contextual understanding, the Transformer layer employs four stacked encoder blocks for hierarchical feature abstraction. Rather than duplicating the functionality of the pre-trained encoder, these additional Transformer blocks operate on the BiLSTM-enhanced representations and focus on modeling long-distance interactions among entities in the downstream sequence labeling task. Each encoder block integrates four core components, as illustrated in Figure 4: a multi-head self-attention mechanism, a feed-forward network (FNN), residual connections, and layer normalization^21^.

Let the input to the *l*-th encoder block be denoted as $\textbf{H}^{(l-1)} \in \mathbb {R}^{n \times d}$, where *l = 1,2,3,4* and $\textbf{H}^{(0)} = \widetilde{\textbf{H}}^{(0)}$ is the normalized output of the BiLSTM layer.

**Multi-head Self-Attention Mechanism:** The query, key, and value matrices are obtained through linear projections:

15 $$\begin{aligned} \textbf{Q}^{(l)} = \textbf{H}^{(l-1)}\textbf{W}_Q^{(l)}, \quad \textbf{K}^{(l)} = \textbf{H}^{(l-1)}\textbf{W}_K^{(l)}, \quad \textbf{V}^{(l)} = \textbf{H}^{(l-1)}\textbf{W}_V^{(l)} \end{aligned}$$

The scaled dot-product attention is computed as:

16 $$\begin{aligned} \textrm{Attention}(\textbf{Q}, \textbf{K}, \textbf{V}) = \textrm{softmax}\left( \frac{\textbf{Q}\textbf{K}^\top }{\sqrt{d_k}}\right) \textbf{V} \end{aligned}$$

where $d_k$ denotes the dimensionality of the key vectors. For the *i*-th attention head, the output is calculated as:

17 $$\begin{aligned} \textrm{head}_i^{(l)} = \textrm{Attention}\left( \textbf{Q}_i^{(l)}, \textbf{K}_i^{(l)}, \textbf{V}_i^{(l)} \right) \end{aligned}$$

The outputs of all heads are concatenated and linearly transformed:

18 $$\begin{aligned} \textrm{MHA}^{(l)} = \textrm{Concat}\left( \textrm{head}_1^{(l)}, \cdots , \textrm{head}_h^{(l)} \right) \textbf{W}_O^{(l)} \end{aligned}$$

The attention output then undergoes a residual connection followed by layer normalization:

19 $$\begin{aligned} \widehat{\textbf{H}}^{(l)} = \textrm{LayerNorm}\left( \textbf{H}^{(l-1)} + \textrm{MHA}^{(l)} \right) \end{aligned}$$

This self-attention mechanism enables the model to dynamically capture global interactions among tokens in the sequence.

**Feed-Forward Network (FNN) Module:** Each encoder block applies a position-wise feed-forward network to the attention output. The FNN consists of two fully connected layers with a ReLU activation function in between:

20 $$\begin{aligned} & \textbf{F}_1^{(l)} = \textrm{ReLU}\left( \widehat{\textbf{H}}^{(l)} \textbf{W}_1^{(l)} + \textbf{b}_1^{(l)}\right) \end{aligned}$$

21 $$\begin{aligned} & \textbf{F}_2^{(l)} = \textbf{F}_1^{(l)} \textbf{W}_2^{(l)} + \textbf{b}_2^{(l)} \end{aligned}$$

The output is then combined with the residual connection:

22 $$\begin{aligned} \widetilde{\textbf{H}}^{(l)} = \widehat{\textbf{H}}^{(l)} + \textbf{F}_2^{(l)} \end{aligned}$$

Final normalization gives

23 $$\begin{aligned} \textbf{H}^{(l)} = \textrm{LayerNorm}\left( \widetilde{\textbf{H}}^{(l)}\right) \end{aligned}$$

The stacked Transformer structure is summarized as

24 $$\begin{aligned} \textbf{H}^{(l)} = \textrm{EncoderBlock}^{(l)}\left( \textbf{H}^{(l-1)}\right) , \quad l = 1,2,3,4 \end{aligned}$$

The final representation $\textbf{H}^{(4)}$ is passed to the distillation and decoding modules.

### Knowledge distillation module

Knowledge distillation (KD) is a technique that transfers knowledge from a teacher model to a student model through softened output distributions^22^. In this framework, the distillation module improves recognition of sparsely labeled entities by transferring token-level soft class distributions from the teacher model. Specifically, the distillation targets are derived from the teacher’s pre-CRF emission logits rather than the final CRF-decoded sequence, so that token-level soft distributions can be aligned consistently between the teacher and student models.

#### Teacher model configuration

The teacher model adopts a simpler TCMBERT-BiLSTM-CRF architecture and is trained on an independent high entity-density annotated dataset with the same annotation criteria and label schema as the student training data. This dataset contains more labeled entity instances per sentence and richer class co-occurrence patterns, which reduce the dominance of the O class and provide denser token-level supervision for learning informative class distributions. Let $\textbf{z}_t^T \in \mathbb {R}^{c}$ denote the pre-CRF logits generated by the teacher model at token position *t*, where *c* denotes the number of entity classes. The teacher logits are taken from the linear emission layer before CRF decoding, so that both teacher and student produce token-level class scores in the same label space. The teacher’s probability distribution is computed using temperature-scaled softmax:

25 $$\begin{aligned} \textbf{P}_t^T = \textrm{softmax}\left( \frac{\textbf{z}_t^T}{\tau }\right) \end{aligned}$$

where *τ* controls the smoothness of the probability distribution. A temperature parameter *τ> 1* produces softer targets, which are more informative for knowledge transfer.

#### Student model learning

The student model receives the contextual feature representation from the feature extraction module and maps it to the label space through a linear classifier. Specifically, the student logits at token position *t* are computed as

26 $$\begin{aligned} \textbf{z}_t^S = \textbf{h}_t^{(4)} \textbf{W}_c + \textbf{b}_c \end{aligned}$$

where $\textbf{h}_t^{(4)}$ denotes the output representation of the final Transformer encoder block, $\textbf{W}_c \in \mathbb {R}^{d \times c}$ is the trainable weight matrix, and $\textbf{b}_c \in \mathbb {R}^{c}$ is the bias vector.

The student probability distribution for distillation is similarly computed with temperature scaling:

27 $$\begin{aligned} \textbf{P}_t^S = \textrm{softmax}\left( \frac{\textbf{z}_t^S}{\tau }\right) \end{aligned}$$

It should be emphasized that the student logits $\textbf{z}_t^S$ are shared by both the knowledge distillation branch and the CRF decoding branch. The temperature-scaled softmax is only used for distillation, whereas the raw logits are directly fed into the CRF as emission scores. In other words, the same token-level emissions are used to compute softened class distributions for distillation and to provide emission scores for CRF-based sequence modeling. This shared design ensures consistency between the distillation objective and the sequence labeling objective.

#### Distillation objective

The discrepancy between the teacher and student distributions is measured using the Kullback–Leibler (KL) divergence:

28 $$\begin{aligned} \mathcal {L}_{\textrm{KD}} = \tau ^2 \sum _{t=1}^{n} \sum _{j=1}^{c} P_{t,j}^{T} \log \frac{P_{t,j}^{T}}{P_{t,j}^{S}} \end{aligned}$$

where $P_{t,j}^{T}$ and $P_{t,j}^{S}$ denote the probabilities assigned by the teacher model and the student model, respectively, to the *j*-th class at token position *t*.

The KL divergence term quantifies the discrepancy between the softened output distributions of the teacher and student models. By minimizing $\mathcal {L}_{\textrm{KD}}$, the student model is encouraged to approximate the class-wise probability structure produced by the teacher model rather than merely fitting hard labels. This enables the student to absorb richer inter-class relational information and latent semantic cues contained in the teacher’s predictions, thereby improving generalization ability, especially in sparse entity recognition scenarios. In implementation, the KD loss is computed only over valid non-padding tokens, and special tokens such as [CLS], [SEP], and [PAD] are excluded from distillation.

The total training objective combines the CRF loss and the distillation loss as

29 $$\begin{aligned} \mathcal {L}_{\textrm{total}} = \alpha \,\mathcal {L}_{\textrm{KD}} + (1-\alpha )\,\mathcal {L}_{\textrm{CRF}} \end{aligned}$$

where $\alpha \in [0,1]$ is the balancing coefficient and $\mathcal {L}_{\textrm{CRF}}$ denotes the negative log-likelihood loss for sequence labeling. In practice, both loss terms are normalized over valid tokens or sequences within each batch before interpolation.

#### Knowledge transfer effect

Through joint optimization, the student model learns both softened semantic guidance from the teacher model and structured labeling constraints from the CRF layer. More specifically, the KD objective regularizes token-level class discrimination, while the CRF objective enforces sequence-level structural consistency.

**Fig. 5:**
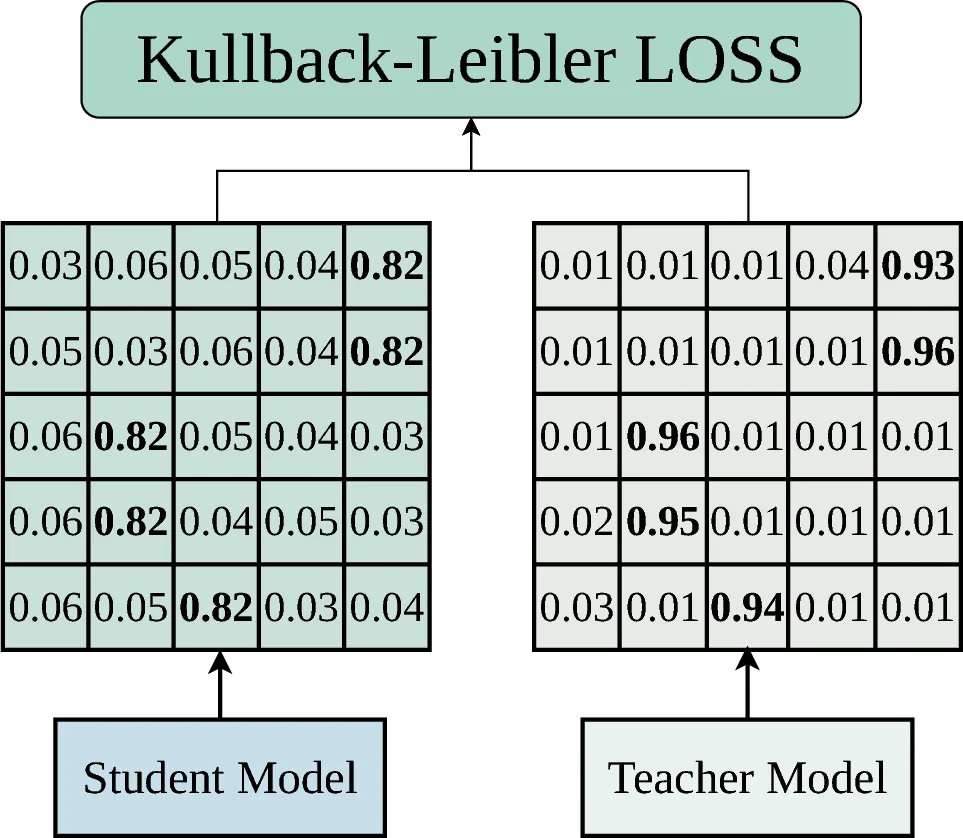
Structure of the knowledge distillation framework.

Notably, the structural characteristics in our framework arise from two sources: the teacher model captures richer semantic patterns from high-density entity corpora, and the CRF layer explicitly models label transition dependencies. This combination allows the distilled knowledge to be aligned with structured sequence prediction. The overall distillation framework is illustrated in Figure 5.

This dual-supervision mechanism may improve robustness and generalization ability of the student model in sparse entity recognition scenarios.

### CRF layer

The Conditional Random Field (CRF) layer^23^ is employed to model dependencies between adjacent labels and to generate globally optimal tag sequences. A trainable state transition matrix $\textbf{A} \in \mathbb {R}^{c \times c}$ is introduced, where $A_{ij}$ denotes the transition score from tag *i* to tag *j*.

For each position *t*, the emission score is directly computed from the student logits produced by the shared classifier:

30 $$\begin{aligned} \textbf{s}_t = \textbf{z}_t^S \end{aligned}$$

where $\textbf{z}_t^S$ denotes the shared token-level emission logits defined in Eq. (26). In this way, the CRF layer and the knowledge distillation module are optimized on the basis of the same emission representation.

Given an input sequence $\textbf{X}$ and a tag sequence $\textbf{y} = (y_1, \dots , y_n)$, the overall sequence score is defined as

31 $$\begin{aligned} S(\textbf{X}, \textbf{y}) = \sum _{t=1}^{n} A_{y_{t-1}, y_t} + \sum _{t=1}^{n} s_{t, y_t} \end{aligned}$$

where $s_{t, y_t}$ denotes the emission score corresponding to assigning label $y_t$ to the *t*-th token. For notational simplicity, the start state is absorbed into the transition formulation.

The CRF layer models the conditional probability of the correct tag sequence as

32 $$\begin{aligned} P(\textbf{y} \mid \textbf{X}) = \frac{\exp (S(\textbf{X}, \textbf{y}))}{\sum _{\tilde{\textbf{y}} \in \mathcal {Y}} \exp (S(\textbf{X}, \tilde{\textbf{y}}))} \end{aligned}$$

where $\mathcal {Y}$ denotes the set of all possible tag sequences for the input sequence.

The training objective minimizes the negative log-likelihood of the gold tag sequence:

33 $$\begin{aligned} \mathcal {L}_{\textrm{CRF}} = -\log P(\textbf{y} \mid \textbf{X}) \end{aligned}$$

During decoding, the Viterbi algorithm is employed to efficiently compute the highest-scoring tag sequence:

34 $$\begin{aligned} \textbf{y}^* = \mathop {\arg \max }_{\textbf{y} \in \mathcal {Y}} S(\textbf{X}, \textbf{y}) \end{aligned}$$

By explicitly modeling inter-label dependencies, the CRF layer reduces invalid tag transitions and improves the consistency of entity boundary detection and category assignment. Within the TBTC architecture, the CRF layer effectively exploits the contextual features extracted by the preceding modules, thereby enhancing the stability and accuracy of TCM entity recognition.

## Experiments

### Experimental settings

#### Datasets

We employ three domain-specific corpora from our constructed TCM knowledge repository for NER model distillation and evaluation: HerbBank, FormulaBank, and CaseBank. HerbBank contains annotated herb-related entities, including nomenclature, medicinal properties (*Xingwei*), and meridian tropism (*Guijing*). FormulaBank records classical TCM prescriptions together with ingredient compositions, therapeutic efficacy, and administration protocols. CaseBank consists of annotated clinical case texts covering multiple entity categories, including symptoms, diseases, formulas, herbs, and syndromes.

To facilitate teacher–student distillation, HerbBank and FormulaBank were merged to form the **TeacherDataset**, whereas CaseBank was used as the **StudentDataset**. The statistics of the resulting datasets are summarized in Table 1. Although the TeacherDataset and StudentDataset were derived from different types of TCM texts, they were constructed under a unified entity schema and the same BIO tagging convention. The entity definitions, boundary rules, and label names were kept consistent across the corpora. For entity categories that appeared in both herb/formula texts and clinical case texts, such as Herb, Formula, Dosage, Property, Channel, Therapy, and Usage, the same semantic scope was applied during annotation. CaseBank additionally contains clinical-context entities such as Disease, Symptom, and Syndrome, which were annotated according to the same entity-boundary principle. Therefore, the teacher–student setting was designed to transfer entity boundary and category-distribution knowledge from high-density herb/formula texts to lower-density and more context-rich clinical case texts, rather than assuming that the two corpora have identical domain distributions.

**Table 1:** Dataset statistics. “Tags” refers to character-level BIO label assignments, rather than entity-level mentions.

Dataset	Type	Train	Val	Test
TeacherDataset	Sentences	111.9k	13.9k	13.9k
	Tags	7,281.6k	919.2k	908.0k
StudentDataset	Sentences	2,916	364	366
	Tags	626.78k	80.97k	75.03k

#### Preprocessing and annotation

During preprocessing, noisy symbols and irrelevant characters were removed to improve text consistency. All texts were then tokenized and annotated under the BIO tagging scheme, where **B**, **I**, and **O** denote the beginning, inside, and outside positions of an entity, respectively.

The annotation was performed according to a predefined entity schema. The schema defines ten entity categories, including Dosage, Channel, Property, Formula, Therapy, Usage, Disease, Symptom, Herb, and Syndrome. During annotation, the complete semantic span of each TCM expression was labeled as one entity. For example, “甘草” was annotated as Herb, “四君子汤” as Formula, “发热” as Symptom, and “脾胃虚寒证” as Syndrome.

For difficult cases, the final entity type was determined according to the contextual function of the term. For example, treatment principles such as “清热解毒” were annotated as Therapy, whereas diagnostic patterns such as “脾胃虚寒证” were annotated as Syndrome. Disease names such as “感冒” were annotated as Disease, while clinical manifestations such as “发热” were annotated as Symptom. When dosage and administration information appeared together, dosage expressions such as “10g” were annotated as Dosage, whereas administration expressions such as “水煎服日一剂” were annotated as Usage. The corpus was annotated by a trained annotator following the predefined entity schema and BIO tagging guideline. To reduce inconsistency, ambiguous cases were checked against the annotation guideline and resolved before finalizing the corpus. Because the original annotation workflow did not include independent double annotation, inter-annotator agreement statistics could not be calculated, which is acknowledged as a limitation of the current dataset construction.

#### Dataset split strategy

Both TeacherDataset and StudentDataset were divided into training, validation, and test sets using an 8:1:1 ratio. The validation set was used exclusively for model selection and hyperparameter tuning, whereas the test set was held out for final evaluation only. To reduce potential sampling bias, dataset splitting was performed with a fixed random seed. In addition, we ensured that the entity-type distribution across the three subsets remained approximately consistent. The entity schema and detailed entity statistics of the StudentDataset are reported in Table 2.

To exclude potential data leakage, the TeacherDataset and StudentDataset were constructed from different source types, and no clinical case document from CaseBank was included in HerbBank or FormulaBank. In addition, duplicate or near-duplicate sentences were removed during preprocessing before dataset splitting. The training, validation, and test sets were separated at the sentence/document level, and the test set was not used during model training, hyperparameter tuning, or teacher-model construction. Although some common TCM entity surface forms, such as frequently used herb or formula names, may naturally appear in both datasets, no identical annotated document or sentence was shared between the teacher and student test data.

**Table 2:** Entity schema and statistics of the StudentDataset.

Entity Name	Category Description	Example	Count
Dosage	Prescription dosage information	10g	260019
Channel	Meridian attribution of prescription	归肝经	1503
Property	Nature and flavor properties of prescription	甘温	3340
Formula	Name of herbal prescription	四君子汤	20234
Therapy	Therapeutic method for syndrome intervention	清热解毒	37403
Usage	Administration method of prescription	水煎服日一剂	22620
Disease	Clinical disease diagnosis	感冒	32898
Symptom	Clinical manifestations of patients	发热	182444
Herb	Name of Chinese medicinal material	甘草	192631
Syndrome	TCM syndrome pattern	脾胃虚寒证	29704

#### Evaluation metrics

Model performance was evaluated using entity-level Precision, Recall, and F1-score based on exact span matching under the BIO scheme:

35 $$\begin{aligned} & \text {Precision} = \frac{TP}{TP + FP} \end{aligned}$$

36 $$\begin{aligned} & \text {Recall} = \frac{TP}{TP + FN} \end{aligned}$$

37 $$\begin{aligned} & ext{F1-score} = \frac{2 \times \text {Precision} \times \text {Recall}}{\text {Precision} + \text {Recall}} \end{aligned}$$

To better assess performance under class imbalance, Macro-F1 and Weighted-F1 were also reported:

38 $$\begin{aligned} & ext{Macro-F1} = \frac{1}{C}\sum _{i=1}^C \text {F1}_i \end{aligned}$$

39 $$\begin{aligned} & ext{Weighted-F1} = \sum _{i=1}^C w_i \text {F1}_i,\quad w_i = \frac{N_i}{\sum _j N_j} \end{aligned}$$

where *TP*, *FP*, and *FN* denote true positives, false positives, and false negatives, respectively; *C* is the number of entity categories; and $N_i$ represents the number of instances in class *i*.

#### Hyperparameter tuning and reproducibility

Hyperparameter selection was performed on the validation set. We tuned the learning rate, batch size, dropout rate, and distillation-related parameters using validation Macro-F1 as the primary selection criterion. The search ranges included learning rates of $\{1\times 10^{-5}, 2\times 10^{-5}, 5\times 10^{-5}, 1\times 10^{-4}\}$, batch sizes of $\{16, 32\}$, dropout rates of $\{0.1, 0.3, 0.5\}$, temperature values of $\{1, 3, 5, 10, 20\}$, and distillation weights $\alpha \in \{0.7, 0.8, 0.9\}$. The final configuration was selected according to the best validation performance while maintaining stable convergence across runs. The final distillation hyperparameters were set to *T=5* and *α =0.9*.

The model used in this study is a pre-trained TCMBERT obtained by further pretraining a BERT model (BertForMaskedLM) on domain-specific corpora. The model checkpoint is located at ./bert. The configuration details are as follows: 12 hidden layers, hidden size of 768, intermediate size of 3072, 12 attention heads, maximum sequence length of 512, attention dropout 0.1, hidden dropout 0.1, layer norm epsilon $1\times 10^{-12}$, GELU activation, vocabulary size 21128, type vocabulary size 2, absolute positional embeddings, and bidirectional encoder. The tokenizer corresponds to this pre-trained checkpoint. The checkpoint configuration also retains the first_token_transform pooler setting.

The BiLSTM hidden size is 256, and the Transformer encoder used in downstream tasks has a hidden dimension of 768 with 12 attention heads. Learning rate scheduling follows a linear decay with 10% warm-up steps. Early stopping monitors validation Macro-F1 with a patience of 7 epochs. Random seeds are fixed for PyTorch, NumPy, and Python random. The CRF layer is implemented using the pytorch-crf library, and preprocessing scripts handle tokenization, sequence padding, and label encoding consistently.

Due to data privacy and institutional restrictions, the datasets are not publicly released at this stage. Access may be considered upon reasonable request to the corresponding author. Tables 3 and 4 summarize the final hyperparameter configuration and computational environment.

**Table 3:** Final model and hyperparameter configuration.

Parameter	Value
Weight decay	0.01
Learning rate	$1\times 10^{-4}$
Batch size	32
Training epochs	100
Gradient clipping	1.0
Optimizer	AdamW
Dropout rate	0.3
Full fine-tuning	True
Early stopping patience	7
BiLSTM hidden size	256
Transformer hidden dimension	768
Transformer attention heads	12
Max sequence length	512
Learning rate schedule	Linear decay
Warm-up proportion	10%
Random seed	42
CRF implementation	pytorch-crf library
Preprocessing scripts	Tokenization, padding, label encoding

**Table 4:** Computational environment.

Component	Specification
IDE	Visual Studio Code
Programming language	Python 3.10
Deep learning framework	PyTorch 2.4.0
Operating System	Linux
GPU	NVIDIA RTX A6000 (48GB VRAM)

#### Statistical significance and robustness

To assess the robustness of the proposed framework, each experiment was repeated over multiple runs with different random seeds, and the mean and standard deviation were reported. Statistical significance was further evaluated by comparing the proposed model with the strongest baseline on entity-level F1-score. Differences were considered statistically significant when *p < 0.05*. In addition, the consistency of performance gains across repeated runs indicates that the observed improvements are not attributable to random initialization alone.

### Experimental results

#### Model performance comparison

To ensure a fair comparison, all Transformer-based models in our experiments used the same TCM-domain pretrained encoder, TCMBERT, as the backbone encoder. The model names in Table 5, such as BERT-CRF and BERT-BiLSTM-CRF, are retained to indicate the commonly used architectural variants in the literature. In implementation, however, the encoder component of these models was replaced with TCMBERT. Thus, “BERT” in the baseline names denotes the architecture family rather than the use of the original general-domain BERT checkpoint. To evaluate the generalization capability of our structured knowledge distillation framework in TCM NER tasks, we conducted comparative experiments with eight representative sequence labeling architectures:

- BiLSTM-CRF (conventional sequence labeling baseline)
- BERT-CRF (baseline)
- BERT-BiLSTM-CRF (bidirectional sequential modeling^24^)
- BERT-BiTCN-CNN (temporal convolutional integration)
- BERT-BiLSTM-MHA-CRF (multi-head attention enhanced^25^)
- Biaffine (span-based baseline)
- RoBERTa-BiLSTM-GlobalPointer (global span detection baseline)
- TBTC (proposed TCMBERT-BiLSTM-Transformer-CRF hybrid architecture)

Table 5 presents the performance metrics on the StudentDataset. It should be noted that the KD setting was applied only to token-level sequence labeling architectures whose output space is compatible with the proposed structured distillation objective. Span-based or pointer-based models, such as Biaffine and RoBERTa-BiLSTM-GlobalPointer, produce different prediction structures and therefore cannot be directly optimized using the same token-level CRF-oriented distillation loss without redesigning the distillation objective.

**Table 5:** Performance evaluation on StudentDataset (%).

Model	KD	Precision	Recall	F1	Macro-F1	Weighted-F1
BiLSTM-CRF	No	70.33±1.27	62.27±1.55	66.03±0.52	49.33±1.52	65.22±0.74
BERT-BiTCN-CNN	No	69.00±1.19	70.71±1.03	69.84±0.60	54.56±2.60	70.00±0.59
BERT-BiLSTM-MHA-CRF	No	68.43±0.92	70.80±0.84	69.59±0.72	53.80±2.23	69.64±0.77
BERT-CRF	No	69.14±1.25	71.41±1.44	70.23±0.57	56.83±0.75	70.39±0.76
BERT-BiLSTM-CRF	No	69.33±1.30	71.78±0.99	70.52±0.69	56.19±2.62	70.64±0.88
Biaffine	No	75.59±0.99	74.87±0.86	75.18±0.65	57.14±1.14	75.55±0.87
RoBERTa-BiLSTM-GlobalPointer	No	74.31±1.03	73.94±0.90	74.09±0.68	57.10±0.97	74.49±0.94
TBTC	No	69.35±0.89	71.37±0.76	70.34±0.57	57.50±0.77	70.65±0.56
BERT-BiTCN-CNN	Yes	72.63±3.10	76.67±0.47	74.57±1.73	63.09±1.50	74.80±1.49
BERT-BiLSTM-MHA-CRF	Yes	72.01±4.23	76.66±0.37	74.21±2.33	63.36±1.91	74.46±2.04
BERT-CRF	Yes	76.13±0.80	76.50±0.36	76.31±0.36	**65.43±1.48**	76.28±0.36
BERT-BiLSTM-CRF	Yes	76.17±1.67	76.23±0.14	76.19±0.79	65.01±0.79	76.16±0.76
TBTC	Yes	**77.36±0.88**	**76.68±0.59**	**77.01±0.31**	65.34±0.40	**76.89±0.27**

**Bold**: Column-wise maximum; Underlined: Column-wise second maximum.*Note*: KD was applied only to token-level sequence labeling architectures with output spaces compatible with the proposed CRF-oriented structured distillation objective. Biaffine and RoBERTa-BiLSTM-GlobalPointer are span-based or pointer-based baselines and are reported as non-distilled reference systems.

Knowledge distillation yielded consistent improvements across all token-level BERT-based models to which the proposed KD objective can be directly applied. For example, the F1-score increased from 69.84 to 74.57 for BERT-BiTCN-CNN, from 69.59 to 74.21 for BERT-BiLSTM-MHA-CRF, from 70.23 to 76.31 for BERT-CRF, and from 70.52 to 76.19 for BERT-BiLSTM-CRF. These observations suggest that the distillation strategy may help transfer informative supervisory signals from the teacher model and improve the discriminative capacity of compatible student models on the constructed dataset.

The proposed TBTC model achieved the best overall results after distillation, with an F1-score of 77.01±0.31. Relative to the non-distilled setting, the model improved by 8.01 percentage points in Precision, 5.31 percentage points in Recall, 6.67 percentage points in F1-score, and 7.84 percentage points in Macro-F1. In particular, the improvement in Macro-F1 suggests that the framework better handles low-frequency entity categories, which are common in TCM NER.

The non-distilled span-based and pointer-based baselines are included as strong reference systems rather than as direct KD-controlled comparisons. A similar trend is observed for Macro-F1 (65.34±0.40 vs. 57.10±0.97), indicating enhanced performance on low-frequency entity categories.This difference can be attributed to the distinct modeling strategies. GlobalPointer focuses on span-level boundary detection, while TBTC adopts a BiLSTM-Transformer-CRF architecture to jointly model local dependencies, global context, and label transitions. Such a design is better suited for handling the sequential complexity and category imbalance in TCM texts.

Furthermore, the small discrepancy between F1-score and Weighted-F1 in the distilled TBTC model suggests that the performance gains were not confined to high-frequency classes alone. Taken together, these results suggest that the integration of BiLSTM and Transformer layers, combined with structured knowledge distillation, can improve overall accuracy and class-level balance on the constructed StudentDataset. Further validation on independent TCM corpora is needed to assess its generalizability.

#### Statistical significance and robustness

To assess the robustness and reproducibility of the proposed framework, each experiment was repeated five times using different random seeds (42, 52, 62, 72, and 82), and the mean and standard deviation were reported. Statistical significance was evaluated by comparing the proposed model with the strongest distilled baseline, BERT-CRF+KD, using paired entity-level F1-scores across repeated runs. Table 6 reports the individual F1-scores for each random seed and the corresponding paired differences. TBTC+KD outperformed BERT-CRF+KD under all five random seeds, with paired improvements ranging from 0.30 to 1.01 percentage points. The mean paired improvement was 0.70 percentage points.

**Table 6:** Per-seed entity-level F1-scores and paired differences between TBTC+KD and BERT-CRF+KD. Values are reported as percentages.

Seed	TBTC+KD F1	BERT-CRF+KD F1	Difference
42	77.14	76.25	+0.89
52	76.75	75.82	+0.93
62	76.56	76.19	+0.37
72	77.38	76.37	+1.01
82	77.23	76.93	+0.30
Mean	77.01	76.31	+0.70

The paired t-test showed that TBTC+KD achieved a higher entity-level F1-score than BERT-CRF+KD (*t(4)=4.646*, *p=0.0097*), where 4 denotes the degrees of freedom rather than the number of runs. The 95% confidence interval of the mean paired improvement was [0.28, 1.12] percentage points. Given the limited number of random seeds, this result should be interpreted as supporting evidence of consistency across random initializations rather than definitive large-sample statistical inference. The detailed statistical test results are presented in Table 7. It should be noted that Table 5 reports population standard deviations over the five repeated runs as descriptive summaries, whereas Table 7 reports sample standard deviations computed with *n-1=4* degrees of freedom, consistent with the paired t-test. This explains the slight difference between the standard deviations reported in the two tables.

**Table 7:** Paired t-test results for entity-level F1-score across five random seeds. SD denotes the sample standard deviation. Mean Diff. and 95% CI are reported in percentage points.

Comparison	Runs	Proposed Model	Baseline Model	Mean Diff.	95% CI	*t*	*p*
TBTC+KD vs. BERT-CRF+KD	5	77.01±0.34	76.31±0.40	+0.70	[0.28, 1.12]	4.646	0.0097

Entity-level or sentence-level bootstrap resampling could further complement the current repeated-run analysis by providing an additional estimate of test-set uncertainty.

#### Category-wise performance comparison with/without distillation

**Fig. 6:**
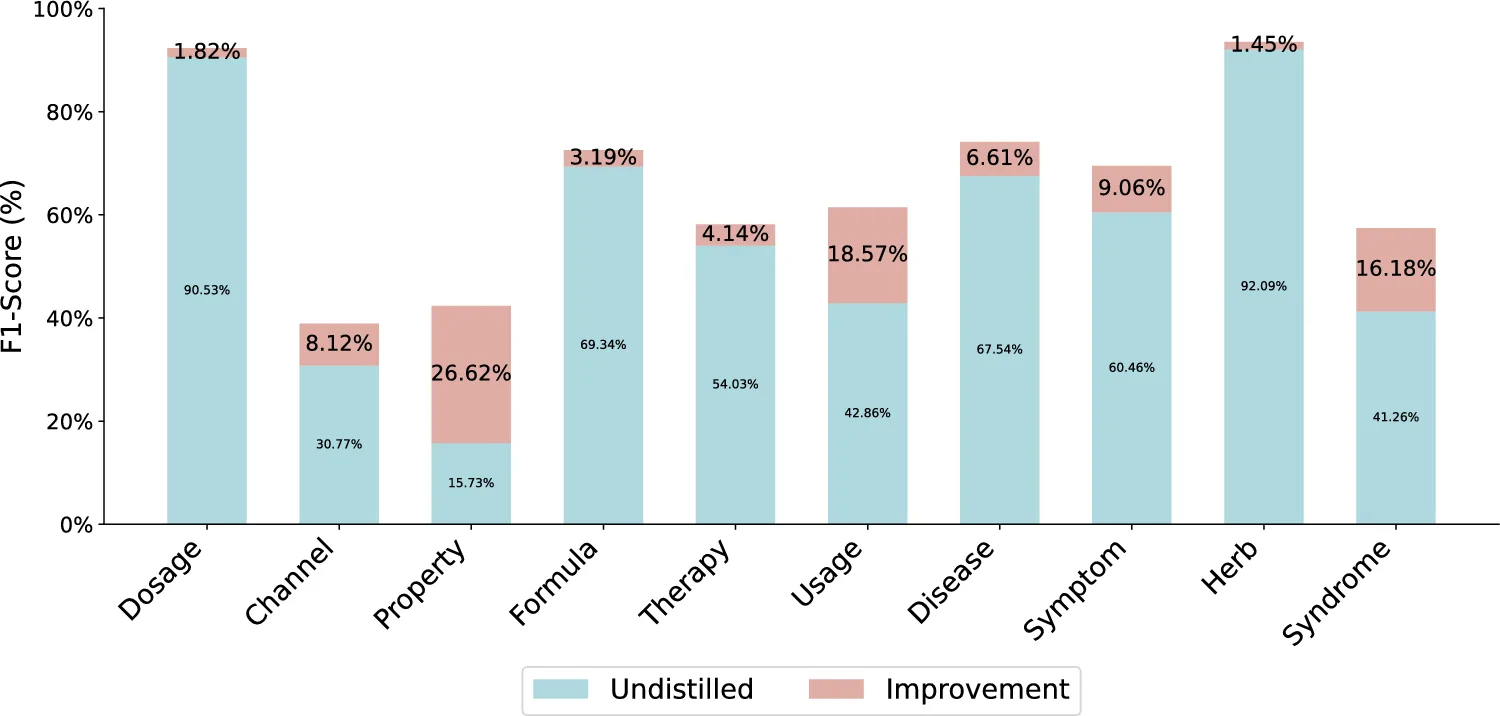
Differential F1 improvement via knowledge distillation across TCM entity categories.

To investigate the differential optimization effects of knowledge distillation across TCM-specific entity categories, we conduct fine-grained performance comparisons on ten characteristic entity types, including dosage, meridian tropism (*Guijing*), and medicinal properties (*Xingwei*). Table 8 reports the per-category Precision, Recall, and F1-score before and after knowledge distillation. Figure 6 further visualizes the F1-score improvements achieved by the proposed framework, illustrating the impact of structured knowledge transfer on different categories.

**Table 8:** Per-category Precision, Recall, F1-score, and test-set entity counts of TBTC with and without knowledge distillation on the StudentDataset. Values of Precision, Recall, and F1-score are reported as percentages. F1 Gain denotes the absolute improvement in F1-score after knowledge distillation, measured in percentage points. Support denotes the exact number of entity instances in the test set.

Entity type	TBTC without KD			TBTC with KD			F1 Gain	Support
	Precision	Recall	F1	Precision	Recall	F1		
Dosage	88.82	92.31	90.53	91.40	93.33	92.35	+1.82	1163
Meridian Tropism (*Guijing*)	25.00	40.00	30.77	29.17	58.33	38.89	+8.12	24
Medicinal Properties (*Xingwei*)	12.50	21.21	15.73	32.14	62.07	42.35	+26.62	56
Formulas	64.19	75.40	69.34	66.89	79.20	72.53	+3.19	148
Treatment Method	55.17	52.94	54.03	55.94	60.58	58.17	+4.14	261
Administration Method	49.32	37.89	42.86	58.90	64.18	61.43	+18.57	73
Disease	68.70	66.41	67.54	71.88	76.55	74.15	+6.61	377
Symptom	58.26	62.83	60.46	70.39	68.66	69.52	+9.06	2736
Herb	92.97	91.22	92.09	93.88	93.20	93.54	+1.45	1095
Syndrome Pattern	41.83	40.70	41.26	54.58	60.62	57.44	+16.18	251

As shown in Table 8 and Figure 6, the *Medicinal Properties* category shows the most substantial improvement (+26.62pp), reaching an F1-score of 42.35% after distillation. Similarly, the *Syndrome Pattern* and *Meridian Tropism* categories exhibit notable gains of 16.18pp and 8.12pp, respectively. In contrast, high-frequency categories such as *Herbs* show only marginal improvement (+1.45pp), and the *Formulas* category demonstrates limited gains (3.19pp) despite a relatively high baseline performance.

These differences are closely related to the imbalanced distribution of entity categories in the StudentDataset. Low-frequency categories, such as *Medicinal Properties*, *Meridian Tropism*, and *Syndrome Pattern*, contain substantially fewer training instances, which limits the effectiveness of conventional supervised learning. Under such conditions, knowledge distillation provides additional soft supervisory signals from the teacher model, enabling the student model to capture more stable inter-class relationships and latent semantic patterns. As a result, the performance improvement is more pronounced in low-frequency categories.

By contrast, high-frequency categories already benefit from sufficient labeled data, and thus gain relatively less from additional supervision. These results indicate that structured knowledge distillation is particularly effective in alleviating data sparsity and enhancing semantic representation for low-frequency and complex entity types in TCM NER.

#### Performance comparison of distillation mechanisms

**Fig. 7:**
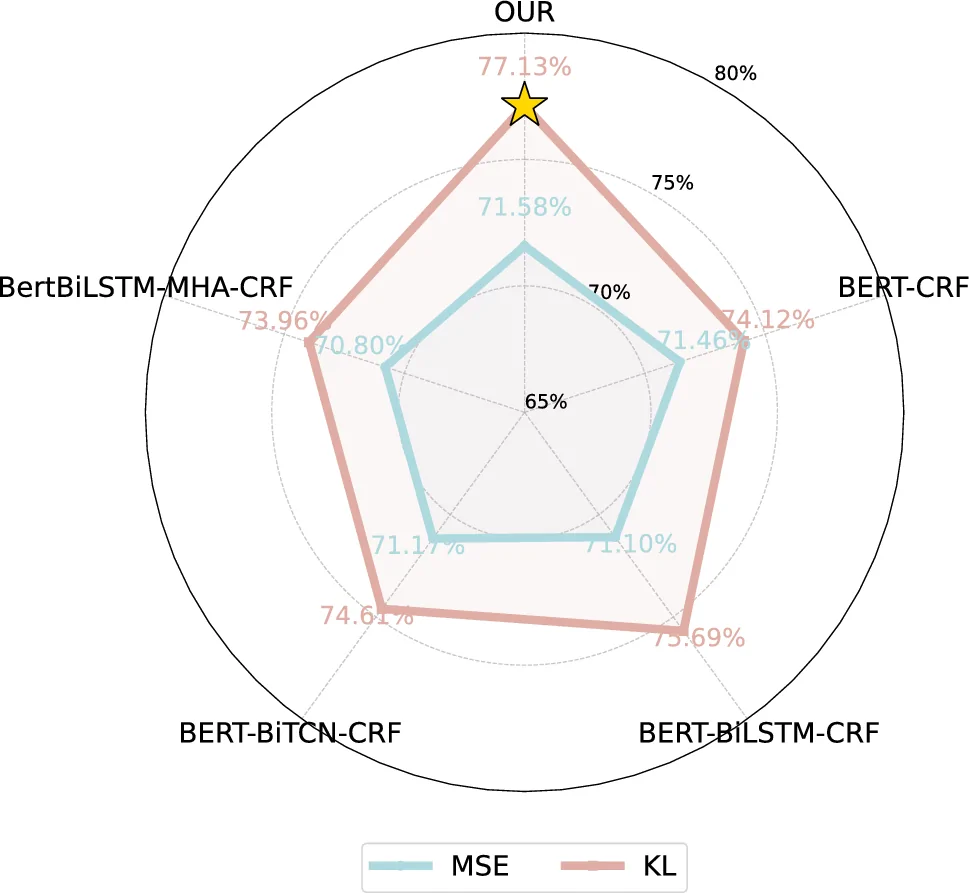
Comparison of KL- and MSE-based distillation across different model architectures.

To examine the effect of different distillation objectives, we compared Mean Squared Error (MSE) and Kullback–Leibler (KL) divergence under the same experimental setting. Five model architectures were evaluated, including the proposed TBTC, BERT-CRF, BERT-BiLSTM-CRF, BERT-BiTCN-CNN, and BERT-BiLSTM-MHA-CRF. As shown in Figure 7, the proposed TBTC model achieved the best performance under both distillation mechanisms, with F1-scores of 71.58 under MSE and 77.13 under KL divergence. By comparison, the baseline models obtained F1-scores ranging from 70.80 to 71.46 under MSE and from 73.96 to 75.69 under KL divergence.

Among the baseline models, BERT-CRF showed comparatively limited performance under the MSE objective, whereas BERT-BiLSTM-CRF achieved the second-highest F1-score under KL divergence, suggesting that bidirectional sequential modeling may be more compatible with distribution-based knowledge transfer. Overall, KL-based distillation consistently outperformed MSE across all evaluated architectures, with an average F1 improvement of 3.12 percentage points. This finding suggests that KL divergence may provide a more suitable objective for structured knowledge transfer in TCM NER.

### Ablation studies

#### Architectural component analysis

To examine the contribution of each component in the proposed framework, we conducted ablation experiments by removing the knowledge distillation (KD) module, the BiLSTM layer, and the Transformer layer, respectively. The results are presented in Table 9. Overall, the full model achieved the best performance across all evaluation metrics, with a Precision of 77.36, a Recall of 76.68, an F1-score of 77.01, a Macro-F1 of 65.34, and a Weighted-F1 of 76.89, indicating that the combination of structured knowledge transfer and hybrid feature modeling is effective for TCM NER.

**Table 9:** Performance comparison of architectural ablations (%).

Model	Precision	Recall	F1	Macro-F1	Weighted-F1
w/o KD	69.35±0.89	71.37±0.76	70.34±0.57	57.50±0.77	70.65±0.56
w/o BiLSTM	75.84±1.26	76.08±0.82	75.95±0.73	64.86±0.84	75.91±0.76
w/o Transformer	76.17±1.67	76.23±0.14	76.19±0.79	65.01±0.79	76.16±0.76
Full Model	**77.36±0.88**	**76.68±0.59**	**77.01±0.31**	**65.34±0.40**	**76.89±0.27**

**Bold**: Column-wise maximum; Underlined: Column-wise second maximum.

Among all ablation settings, removing the KD module led to the most substantial performance degradation. In comparison with the full model, Precision, Recall, and F1-score decreased by 8.01, 5.31, and 6.67 percentage points, respectively, while Macro-F1 dropped by 7.84 percentage points. This result suggests that knowledge distillation makes a substantial contribution not only to overall recognition accuracy but also to class-balanced performance, especially for low-frequency entity categories.

The removal of the BiLSTM layer also resulted in a clear decline in performance, reducing the F1-score from 77.01 to 75.95 and the Macro-F1 from 65.34 to 64.86. This finding indicates that BiLSTM remains important for modeling local sequential dependencies and preserving contextual continuity in entity recognition. Similarly, eliminating the Transformer layer reduced the F1-score to 76.19 and the Macro-F1 to 65.01, suggesting that global contextual modeling also contributes to performance improvement. Compared with the BiLSTM ablation, the performance drop caused by removing the Transformer layer was slightly smaller, but still consistent across all reported metrics.

Taken together, these results show that the KD module contributes the largest performance gain, while the BiLSTM and Transformer layers provide complementary benefits for local and global context modeling, respectively. The superior performance of the full model therefore supports the effectiveness of integrating hierarchical representation learning with structured knowledge distillation in TCM named entity recognition.

#### Hyperparameter analysis

**Fig. 8:**
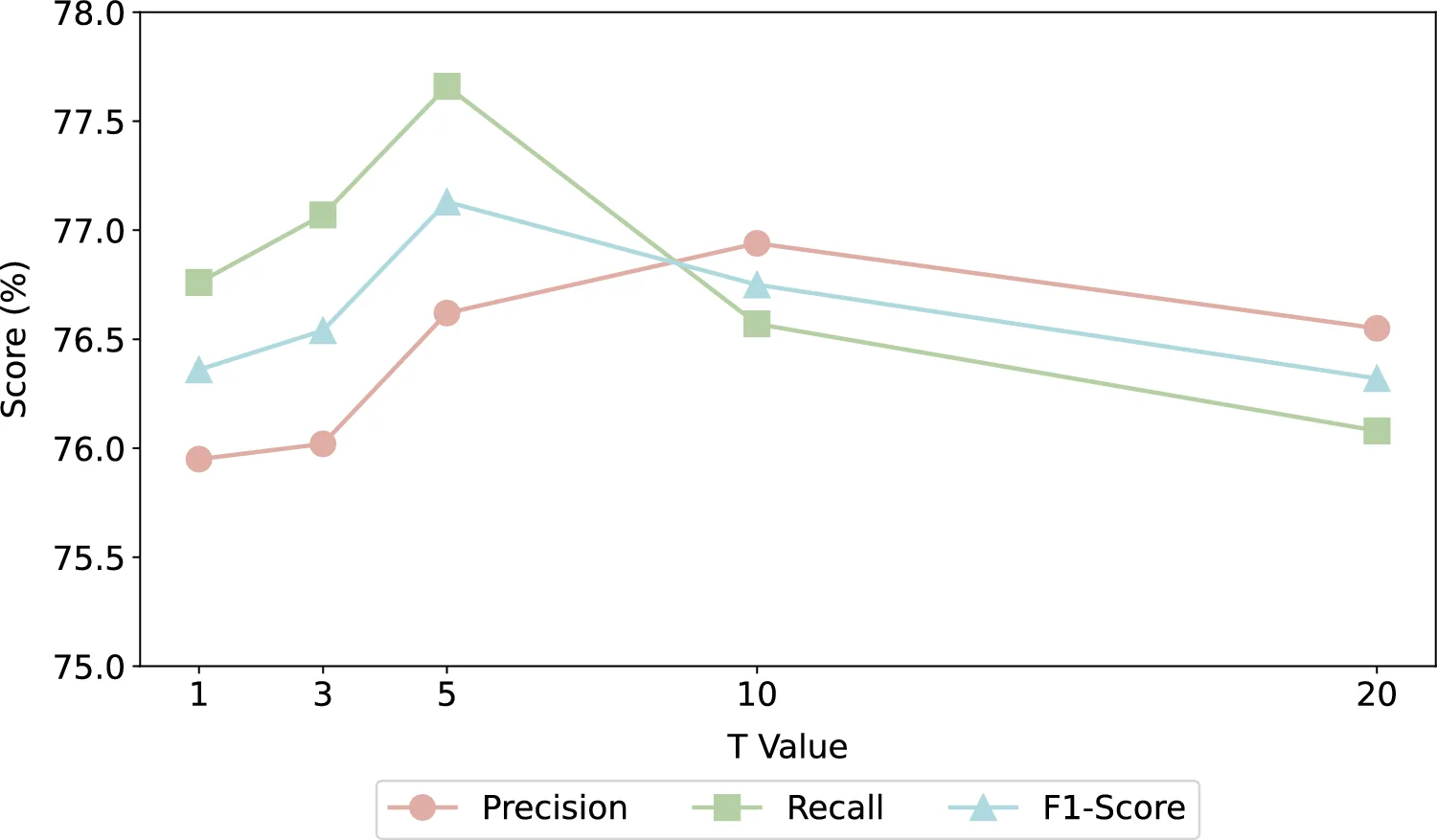
Effect of temperature parameter T on model performance.

To examine the impact of key hyperparameters in the distillation framework, we analyze the role of the temperature parameter *T*, as shown in Figure 8. A moderate temperature improves performance by smoothing the prediction distribution and alleviating over-confident decisions caused by ambiguous entity boundaries. In contrast, excessively high temperatures lead to over-smoothed distributions, which weaken discriminative ability, particularly for low-frequency entities.

**Fig. 9:**
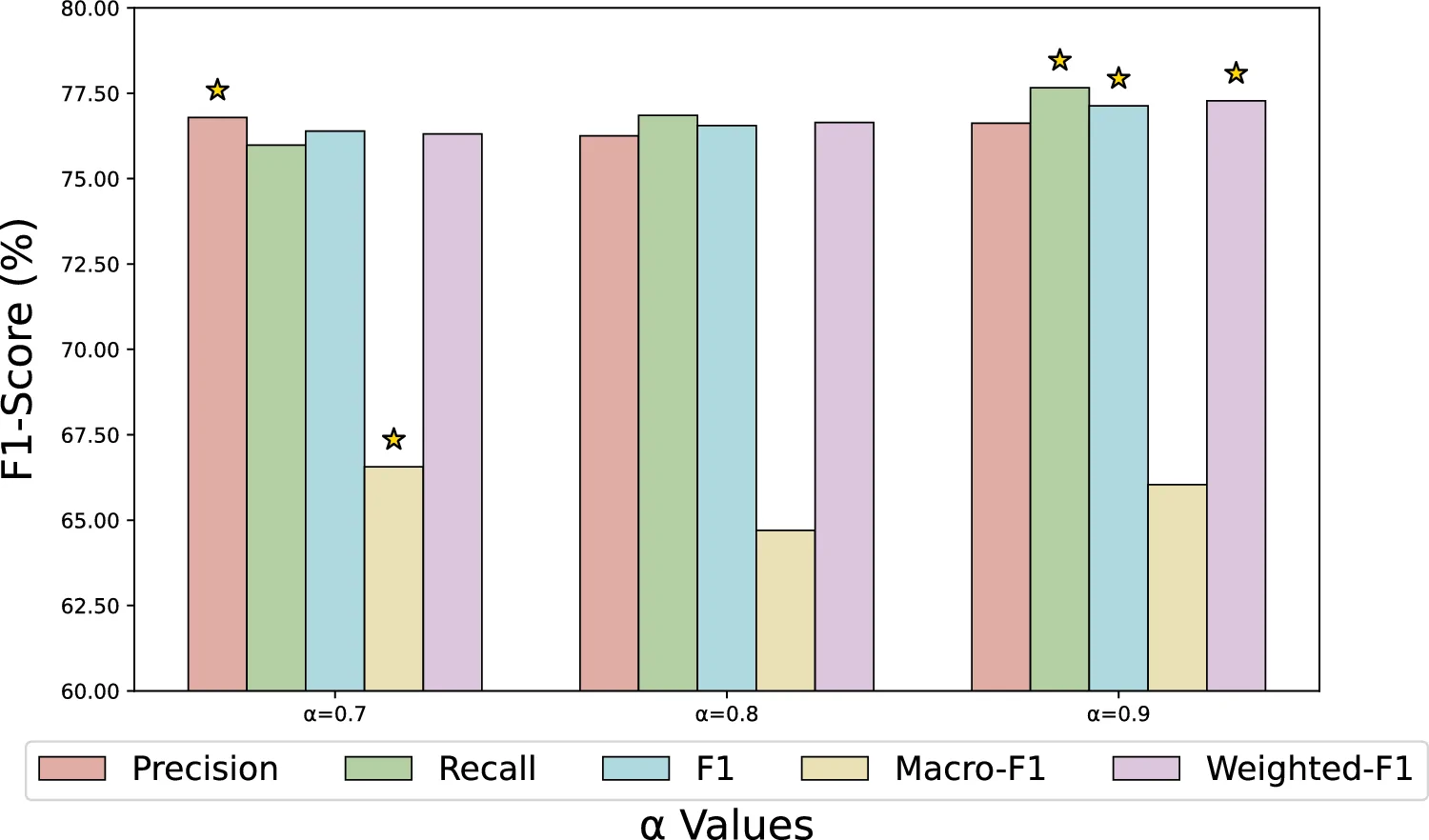
Differential *α* value Effects Comparison.

As illustrated in Figure 9, increasing *α* enhances the contribution of teacher knowledge, enabling the model to better capture implicit semantic relationships and long-tail patterns. However, smaller values of *α* shift the optimization focus toward raw annotations, resulting in reduced sensitivity to low-frequency entities and a bias toward high-frequency patterns.

### Representation analysis

**Fig. 10:**
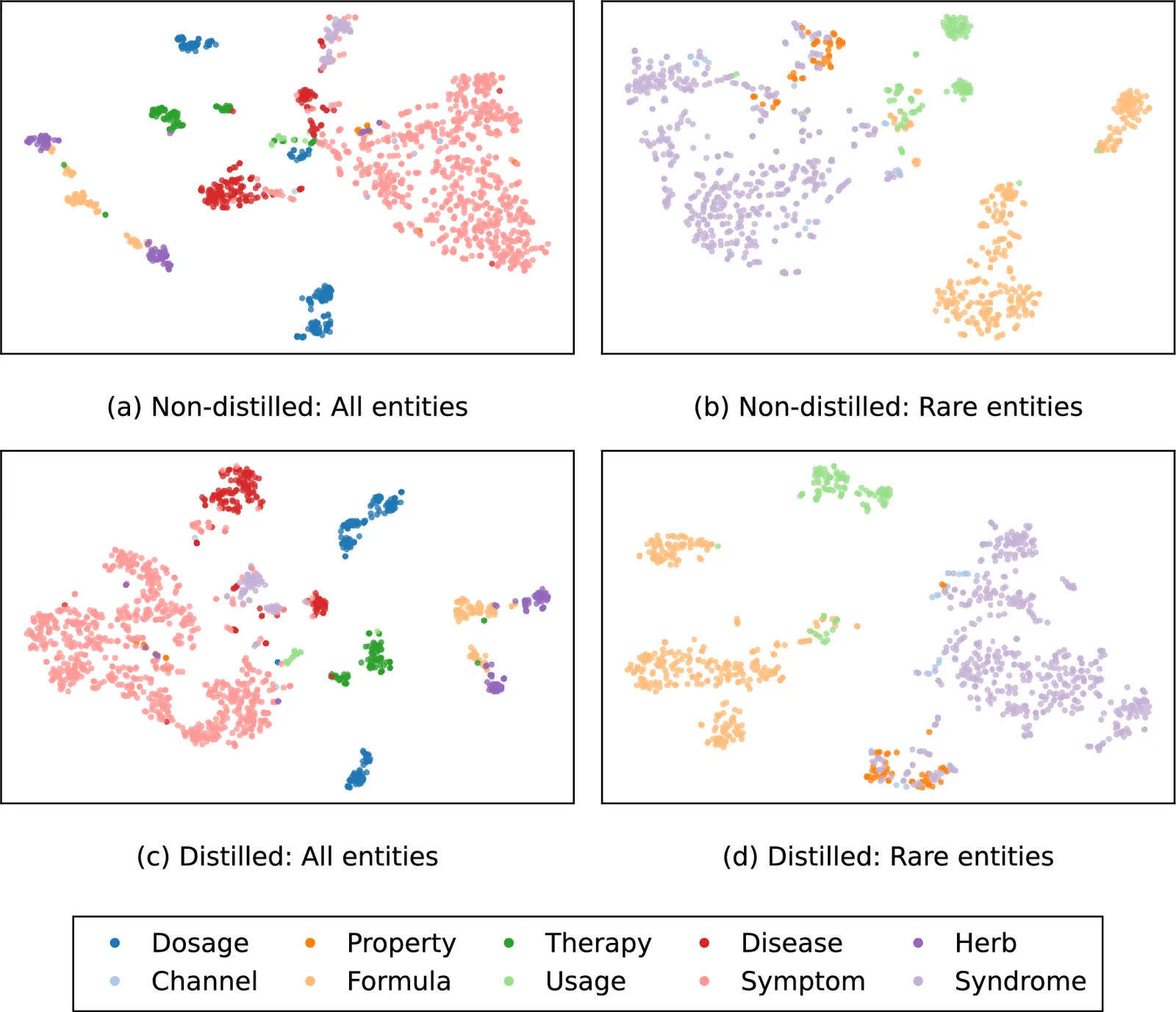
Visualization of token-level entity representations learned by the non-distilled and distilled models using t-SNE on the StudentDataset test set. Subfigures (**a**) and (**b**) show all-entity and low-frequency-entity representations of the non-distilled model, respectively, while subfigures (**c**) and (**d**) show the corresponding results for the distilled model. Different colors denote different entity categories.

To examine the impact of knowledge distillation on representation learning, we visualize token-level entity representations from the StudentDataset test set using t-SNE, as shown in Figure 10. Only entity tokens were included; t-SNE was performed with perplexity = 30, 1000 iterations, PCA dimension = 50, and random seed = 42. For visualization, we sampled up to 1500 entity tokens for all-entity plots and up to 1000 tokens from the five least frequent entity categories with fewer than 100 instances.

Both models show category-aware structures, where tokens of the same entity type tend to form local clusters, suggesting that entity-related semantic information is captured to some extent. Nevertheless, the two models differ in cluster compactness and inter-class separation. For all entity tokens, the distilled model shows clearer boundaries and reduced overlap among several categories, suggesting a more structured representation space. This pattern becomes more evident for low-frequency entities, where the non-distilled model shows more dispersed distributions with noticeable inter-class mixing, while the distilled model forms relatively more compact clusters with improved separability. Overall, these observations suggest that knowledge distillation may improve the organization and discriminability of the representation space, particularly for underrepresented entity categories.

## Discussion

The structured knowledge distillation framework presented in this study showed improvements in NER performance for TCM on the constructed StudentDataset, particularly in the recognition of low-frequency entities, such as medicinal properties (*Xingwei*) and syndrome patterns. The observed F1-score improvements, with gains exceeding 26 percentage points in these categories, suggest that transferring soft label information from a teacher model can be beneficial under the current teacher–student corpus setting. This approach leverages structured knowledge learned from high-density data to alleviate, to some extent, the challenges posed by sparse annotations in TCM texts.

Despite these improvements, certain entities, such as meridian tropism (*Guijing*), continue to present challenges, as evidenced by a relatively low F1-score of 40.47%, compared to the 72.53% achieved for formulas. This limitation can be attributed to several factors. First, meridian entities are underrepresented in the training data, leading to insufficient supervision. Second, these entities are frequently expressed in abstract or metaphorical terms within classical TCM texts, where explicit boundaries may be absent, and their meaning often overlaps with symptoms or therapeutic concepts. Additionally, while the framework may improve token-level discrimination and sequence-level consistency, it does not explicitly model higher-level structures, such as span-level representations or entity-level interactions, which are important for disambiguating complex expressions.

The proposed knowledge distillation strategy is most suitable when the teacher corpus has high entity density, reliable annotations, and a label schema consistent with the student corpus. In this study, HerbBank and FormulaBank were used as the TeacherDataset because they contain dense herb- and formula-related entity mentions and follow the same BIO tagging convention and entity-boundary principles as CaseBank. Therefore, the current teacher–student design aims to transfer entity-boundary and category-distribution knowledge from high-density herb/formula texts to clinical case texts, rather than assuming that the two corpora have identical domain distributions.

The sensitivity analysis suggests that model performance is sensitive to parameter settings, particularly the values of *T=5* and *α =0.9*, when low-frequency entities constitute less than 15% of the training data. This indicates that the effectiveness of the distillation process may depend on both the structural quality of the teacher model and the distribution of training data.

However, if the teacher corpus is insufficient, noisy, weakly annotated, or substantially mismatched with the target student corpus, the teacher model may introduce biased supervisory signals and potentially lead to negative transfer. Since this study did not conduct additional robustness analysis under low-quality or insufficient teacher-data settings, the findings should be interpreted within the current setting of a consistently annotated TCM teacher–student corpus. Future work will further evaluate the robustness of the proposed framework under different teacher-data scales, annotation qualities, and cross-domain transfer scenarios.

To address these limitations, several avenues for future improvement are identified. The implementation of class-balanced training techniques or the adoption of data augmentation strategies could help mitigate the effects of data sparsity, particularly for underrepresented entity types. Furthermore, incorporating span-level supervision and modeling structural dependencies could improve boundary detection for complex entity expressions. The integration of domain-specific knowledge, such as meridian topology or acupuncture-related ontologies, may further enhance the model’s ability to represent and resolve ambiguities in challenging TCM concepts.

Although the proposed TBTC model achieved improved NER performance on the constructed StudentDataset, it introduces additional computational cost due to the combination of TCMBERT, BiLSTM, Transformer, and CRF layers. The current study primarily focuses on recognition performance and does not systematically evaluate deployment-oriented indicators, such as inference speed, GPU memory consumption, model size, or throughput. Therefore, the practical deployment applicability of the model should be interpreted with caution.

Future work will further investigate lightweight optimization strategies for practical TCM text-processing scenarios. Possible directions include distilling the current TBTC model into a smaller student model, applying parameter pruning or quantization, reducing the number of Transformer layers, and exploring lightweight encoder architectures. In addition, future evaluations will include inference latency, memory usage, and throughput under realistic deployment environments to better assess the trade-off between accuracy and efficiency.

Beyond these technical considerations, the proposed framework may be useful for TCM informatics tasks. Accurate entity recognition is an important step in structured information extraction from large-scale TCM texts and may support the development of TCM knowledge bases. Such knowledge bases could further facilitate information retrieval, knowledge organization, and downstream relation extraction. However, the present study did not evaluate downstream clinical decision-support performance or validate the model on independent external TCM corpora. Therefore, any claims regarding clinical decision-support applications should be regarded as potential future directions rather than conclusions directly supported by the current experiments.

In conclusion, the structured knowledge distillation framework improved TCM NER performance on the constructed StudentDataset, especially for several low-frequency entity categories. Nevertheless, the current findings are limited to the datasets and experimental settings used in this study. Further validation on independent TCM corpora and downstream application scenarios is required to assess the generalizability and practical utility of the proposed approach.

## Conclusion

To address key challenges in TCM NER, including sparse entity distributions, term polysemy, and poor recognition of low-frequency entities, this study proposes a TCMBERT-BiLSTM-Transformer-CRF framework with structured knowledge distillation. In addition, a multi-source TCM corpus containing herbological texts, formulas, and medical case records was constructed to support model training and evaluation.

Experimental results on the constructed StudentDataset suggest that the proposed framework was associated with improved TCM NER performance under the current experimental setting. The distilled TBTC model achieved a Precision of 77.36%, a Recall of 76.68%, and an F1-score of 77.01%. Compared with its non-distilled counterpart, the model improved by 8.01 percentage points in Precision, 5.31 percentage points in Recall, 6.67 percentage points in F1-score, and 7.84 percentage points in Macro-F1. The results also suggest that knowledge distillation may improve the performance of comparable BERT-based models and may be particularly beneficial for low-frequency entity categories in the current setting. In category-level evaluation, the *Medicinal Properties* category achieved the largest gain, with an F1-score improvement of 26.62 percentage points after distillation.

Ablation experiments further indicate the contribution of each component in the proposed architecture. Removing the knowledge distillation module led to the largest performance drop, while removing the BiLSTM or Transformer layer also reduced overall performance, suggesting that structured knowledge transfer and hybrid contextual modeling both contribute to performance improvement in the current setting. In addition, statistical significance testing showed that TBTC+KD achieved a higher entity-level F1-score than BERT-CRF+KD (*t(4)=4.646*, *p=0.0097*), which provides preliminary supporting evidence of consistency across random initializations, although the limited number of random seeds should be considered when interpreting this result.

Despite these encouraging results, the model still shows limitations in recognizing certain complex and low-resource entity types, such as meridian tropism, whose expressions are often abstract and context-dependent in classical TCM texts. Moreover, the current findings are based on the constructed StudentDataset and have not yet been validated on independent external TCM corpora or downstream clinical decision-support tasks. Future work will focus on improving boundary detection for complex entities, incorporating richer domain knowledge, evaluating the framework on independent TCM datasets, and further assessing its utility in downstream TCM information extraction and knowledge organization scenarios.

## Data Availability

The three corpora used in this study, namely HerbBank, FormulaBank, and CaseBank, are not publicly available because they include clinical records and other materials containing potentially sensitive information. Although identifying information was removed during data processing, unrestricted public release may still pose privacy and data-governance concerns. For this reason, the datasets are not deposited in a public repository. To support research transparency and reproducibility, the annotation scheme, data preprocessing procedure, model settings, and training details are described in the manuscript. Researchers interested in accessing the datasets for academic purposes may submit a reasonable request to the corresponding author. Requests will be considered on a case-by-case basis and, where appropriate, access may be granted under conditions that protect data privacy and comply with institutional requirements. The code, model configurations, and experimental settings used in this study are available from the corresponding author upon reasonable request.
